# Bone tuberculosis simulating an exostosis

**DOI:** 10.11604/pamj.2014.17.173.4076

**Published:** 2014-03-07

**Authors:** Youssef El Bir, Monsef Boufettal

**Affiliations:** 1 Orthopedic Surgery Department of Ibn Sina Hospital, Rabat, Morocco

**Keywords:** Bone, tuberculosis, exostosis

## Image in medicine

A 32-year old male presented with pain and swelling involving the distal third of the left thigh. He initially noticed a small swelling which eventually increased in size. Patient also had low grade fever without any diurnal variation. There was no past history of antitubercular treatment as well as there was no family history of tuberculosis. There was history of weight loss and loss of appetite. Physical examination revealed an 80 × 40 mm, painful and firm mass of the left lower extremity which had gradually worsened (A). He denied any trauma to the site. Motor and sensory examinations were normal and there was no lymphadenopathy noted in the inguinal areas. No other acute symptoms were reported by the patient. The radiography of the left thigh was objectified a bony overgrowth with well-defined margins making evoke an exostosis (B), CT scan showed a 30 mm × 48 mm ×77 mm pseudo tumor with irregular margin associated with calcifications (C). Erythrocyte sedimentation rate (ESR) 33mm, CRP 80mg/l typically suggestive of infection. The bacteriological examination was positive for Mycobacterium tuberculosis. Histological examination showed the presence of an epitheloid giant cell granuloma with caseous necrosis. Based on the culture and histopathology, patient was started on antitubercular therapy with isoniazid, rifampicin, ethambutol and pyrazinamide. At 6 months follow up, we note a good clinical improvement.

**Figure 1:**
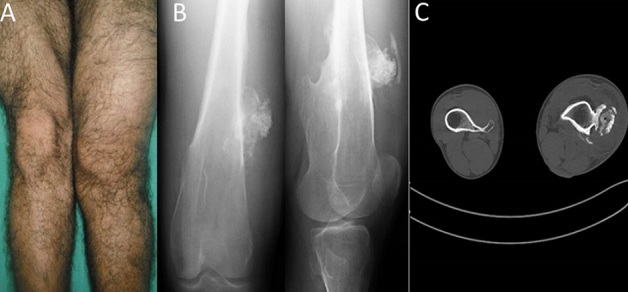
A)Clinical picture showing swelling in the distal third of the left thigh; B) Radiography of the left thigh showing a bony overgrowth; C) CT scan showing pseudo-tumoral aspect of tuberculosis associated with calcifications

